# Modeling Interface Damage with Random Interface Strength on Asphalt Concrete Impervious Facings

**DOI:** 10.3390/ma17133310

**Published:** 2024-07-04

**Authors:** Hui Peng, Nanxuan Qian, Desheng Yin, Wei Luo

**Affiliations:** 1 Hubei Key Laboratory of Hydropower Engineering Construction and Management, China Three Gorges University, Yichang 443002, China; hpeng1976@163.com (H.P.); nx.qian@ctgu.edu.cn (N.Q.); deshengyin@163.com (D.Y.); 2 College of Hydraulic & Environmental Engineering, China Three Gorges University, Yichang 443002, China

**Keywords:** adhesion–decohesion damage, Mazars damage, cohesive zone model, impervious facings

## Abstract

Asphalt concrete impervious facings, widely adopted as the impervious structures for rockfill dams and upper reservoirs in pumped storage power stations, typically have a multilayer structure with a thin sealing layer, a thick impervious layer, and a thick leveling bonding layer. The properties of the interfaces between these layers are crucial for the overall performance of the facings. This paper develops a model to investigate the complex interface damage behavior of the facing under static water pressure and gravity. The model considers two damage origins: one is the interface adhesion–decohesion damage, which is described by the cohesive zone model (CZM) combined with the Weibull-type random interface strength distribution, and the other is the bulk damage of each layer, described by Mazars’ model. Primarily, a comparison between numerical simulation and indoor direct shear tests validates the reliability of the CZM for the asphalt concrete layer interface. Then, the damage distribution of the two interfaces is simulated, and the characteristics of the interface stress are analyzed in detail. The interface shear stresses of the ogee sections, which have different curvatures, all show an interesting oscillation between the thin sealing layer and the impervious layer, and the interface damage at this interface exhibits high heterogeneity. Furthermore, tension stress exists in the local zones of the ogee section, and the damage in this section is significantly greater than in other parts of the facings.

## 1. Introduction

Asphalt concrete impervious facings have been widely used as seepage control structures in embankment dams since the 19th century [1] in Europe. Today, simple-section asphalt impervious facing has been extensively applied in pumped storage hydropower projects in China [2] due to its many advantages, such as the ease of construction, adaptability to settlement, and resistance to freeze–thaw [3]. This layered structure consists of a thin sealing layer, a thick impervious layer, and a thick leveling bonding layer. The interlayer properties are essential to the performance of the facings [4]. Under the combined effects of internal and external stresses, the interfaces between the layers may experience various forms of damage, such as adhesion and decohesion [5]. Subsequently, water vapor may enter through the damaged interfaces, causing blistering, cracking, and wrinkling, ultimately leading to the failure of the impervious system [6]. Therefore, the damage degree at the bonding interface is an essential indicator for assessing the performance of impervious facings [7].

It is worth noting that scholars have extensively studied the interface damage mechanism of asphalt mixtures and the factors influencing their pavement performance [8]. Indoor tests have shown that the interlayer bonding strength of asphalt concrete in road engineering is related to factors such as the temperature and the amount of asphalt sprayed [9,10]. These results are useful for reference purposes in impervious facings. However, it is often difficult to use them directly to evaluate the layer interface strength in facings, mainly due to limitations in the sample size and structure, as well as the experimental conditions. Specifically, the thickness of the sealing layer is too thin for direct-shear tests and too thick to be suitable for indentation and scratch tests [11,12].

In light of the above difficulties in relation to experiments, numerical methods such as the discrete element method (DEM) and the finite element method (FEM) are powerful tools for investigating the layer interface damage and seepage mechanism of impervious facings [13]. DEM models the behavior of materials through particle discretization and possesses prominent advantages in treating the complex contact behavior between many particles [14,15,16,17,18]. However, the key problem is that the parameters of the contact model, which is an important cornerstone of DEM, such as the spring stiffnesses, friction coefficients, and viscosity coefficients, are difficult to calibrate through conventional experiments [19]. In contrast, FEM is a method used to solve the physical problems of continuous media by discretizing them into a finite number of elements and only needs several parameters that have clear physical definitions and can be easily obtained by experiments [15]. Furthermore, thanks to the development of the CZM based on interface and damage mechanics [16], FEM can well describe the crack initiation and propagation process in brittle and quasi-brittle materials, and also the interfacial damage behavior, including sliding, debonding, and shear failures, in composite materials and structures [20]. Therefore, FEM combined with the CZM is an effective approach to establishing a practical and reliable model of the interface mechanics of impervious facings.

When the FEM combined with the CZM is used to analyze the interface mechanical contact problems of impervious facings, it is almost always inappropriate to assume perfect bonding at the interfaces, as they are affected by many unavoidable factors, such as the inhomogeneous bonding strength from construction defects and physical property deviations [20,21]. The inhomogeneous bonding strength significantly affects the adhesion and decohesion between layers of these facings and can be represented by introducing a random distribution of the interface layer strength [22,23]. From previous research [24,25], the Weibull distribution model can describe the randomness of the interface strength in composite materials. Note that neglecting this randomness can lead to discrepancies between simulation results and actual performance [26,27]. The decision to use COMSOL Multiphysics 6.2^®^ for the simulation is based on several considerations. Although it involves a large computational load when dealing with mechanical contact, it ensures precision and has computational advantages in modeling interfacial damage.

In addition, asphalt concrete impervious facings belong to the so-called film–substrate systems, and the curvature of the ogee sections strongly influences their mechanical behavior. The outer sealing layer of the simple-section impervious facing is formed by brushing SBS-modified asphalt mastic, which then solidifies onto the inner layer. This layer is rather thin, only about 2 mm, and very soft, with a relatively low stiffness; in comparison, the other two layers are relatively thick, about 8–10 cm, with much higher stiffness [28,29,30]. As indicated by previous studies [31,32], the initial curvature plays a key role in the wrinkling, instability, and post-buckling of the surface thin films in these systems. Accordingly, the curvature of the ogee sections will strongly affect the mechanical performance of impervious facings. The potential interface wrinkling between the sealing and impermeable layers may be a vital incentive for the progressive damage and leakage of facings. Therefore, elucidating the instability and failure mechanisms of the interlayer interfaces in the impervious facings with different curvature ogee sections is of great significance.

In this study, we have developed an interfacial mechanical behavior model based on two hybrid damage models. Utilizing the results from the indoor interface direct shear tests obtained from Ref. [11], we have validated the applicability of the proposed interfacial damage model for asphalt mixture under direct shear conditions. This model not only takes into account the micro-mechanisms of material damage but also specifically considers the impact of random strength on the adhesion–debonding damage. Furthermore, we have extended this model to asphalt mixture multi-layer impervious facings by considering the bi-parameter Weibull random modulus, and we have explored the shear stress and adhesion–decohesion damage distribution characteristics on the double-layer interface of the anti-arc segment under different curvature conditions, as well as the influence of the Weibull modulus parameters on the interfacial damage evolution process. In the construction of impervious facings, the material construction techniques, quality fluctuations, and the uncertainty of the adhesion forces between each layer of the sealing layer, impervious layer, and leveling bonding layer are three key factors that affect the structural performance. The Weibull random modulus distribution can effectively simulate the randomness of these factors, thereby helping to assess and predict the impact of the material strength and construction quality on the structure, as well as the risk of adhesion failure.

## 2. Model Specification and Simulation Method

### 2.1. Damage Model

#### 2.1.1. Interface Adhesion Mechanical Damage

The CZM has been well developed and widely applied in the simulation of the cracking of quasi-brittle materials, including concrete-like materials, and it is especially useful for the interface [33]. The model assumes the existence of a small fracture process zone at the crack tip region, and the cohesive forces prevent the separation of the crack interface. Commonly, the stress-displacement function is generally used to describe the crack behavior, so a displacement-based damage model is appropriate, in which the damage variable $D_{disp}$ is positively correlated with the displacement variable. Due to the mixed mode of fracture patterns, including modes I and II, a mixed mode displacement $u_{m}$ is introduced as the norm of the jump vector of displacement and can be expressed as Equations (1) and (2) [34]:

(1) $$u_{m}=\left\|u\right\|.$$

(2) $$D_{disp}=\left\{\begin{array}{ll} 0 & \;u_{m,\max}<u_{0m}\\ \min[F^{-1}(u_{m,\max}),1] & \;u_{m,\max}\geq u_{0m}\; \end{array}\right.,$$

The variable $D_{disp}$ represents the damage variable. During the shearing process, the damage variable increases, leading to interface softening until the interface undergoes ultimate failure. Before calculating $D_{disp}$, it is necessary to compute the maximum value um in the loading history within the finite element. This expression involves the damage evolution function $F^{-1}$ and the initial damage variable $u_{0m}$, where $u_{0m}$ is the beginning of the definition of damage, or what can be called the damage threshold. Essentially, it involves solving the equation for the stress vector f, where the CZM can be seen as a regularization of linear elastic fracture mechanics, distributing the energy release rate across the entire interface region. This distribution leads to a progressive process of delamination and damage at the interface rather than to sudden fracture at a single point. The stress vector is defined as $f=(1-D_{disp})Ku$, where K is the stiffness matrix. Here, $F^{-1}$ represents the softening phase of the traction–separation law F and can be defined as [34]:

(3) $$F^{-1}(u_{m,\max})=\frac{u_{\mathrm{fm}}}{u_{m,\max}}(\frac{u_{m,\max}-u_{0m}}{u_{\mathrm{fm}}-u_{0m}}),$$

where $u_{\mathrm{fm}}$ is defined as the critical displacement for linear mixed-mode fracture $u_{0m}$ and can be written by:

(4) $$u_{0m}=u_{0t}u_{0s}(\frac{u_{m}^{2}}{{\langle u_{I}\rangle }^{2}u_{0s}^{2}+u_{\mathrm{II}}^{2}u_{0t}^{2}}{)}^{1/2}.$$

The values of $u_{0t}=\frac{\sigma_{t}}{k_{n}}$ and $u_{0s}=\frac{\sigma_{s}}{k_{t}}$ are calculated based on the interfacial tensile strength $\sigma_{t}$, the normal stiffness $k_{n}$, the interfacial shear strength $\sigma_{s}$, and the tangential stiffness $k_{t}$.

It should be noted that the failure displacement of the mixed mode is determined using the Benzeggagh–Kenane criterion, which is crucial for solving the interfacial shear stress and can be defined by [35]:

(5) $$G_{\mathrm{ct}}+(G_{\mathrm{cs}}-G_{\mathrm{cs}})(\frac{G_{\mathrm{II}}}{G_{1}+G_{\mathrm{II}}}{)}^{a}=G_{I}+G_{\mathrm{II}}.$$

According to the equation above, $G_{\mathrm{ct}}$ and $G_{\mathrm{cs}}$ are the energy release rates for the tension and shear mode, respectively, and α is the mixed-mode index. Based on the equation above, the failure displacement $u_{\mathrm{fm}}$ can be defined as [35,36]:

(6) $$u_{\mathrm{mf}}=\left\{\begin{array}{ll} \frac{2u_{m}^{2}}{u_{m0}(k_{n}u_{I}^{2}+k_{t}u_{\mathrm{II}}^{2})}[G_{\mathrm{ct}}+(G_{\mathrm{cs}}-G_{\mathrm{ct}})(\frac{\frac{k_{t}{\;\;}^{2}}{k_{n}}u_{\mathrm{II}}^{2}}{u_{I}^{2}+\frac{k_{t}}{k_{n}}u_{\mathrm{II}}^{2}}){]}^{a} & \;u_{I}>0\\ \frac{2G_{\mathrm{cs}}}{\sigma_{s}} & u_{I}\leq 0 \end{array}\right..$$

The computational model in this study is subject to geometric nonlinearity during the simulation. To prevent errors related to surface embedding, it is necessary to define a search criterion for the target boundary in relation to the source boundary. To achieve this, a penalty function is introduced to restrict unreasonable gaps. This function can be expressed by the following equation [34]:

(7) $$T_{n}=\left\{\begin{array}{ll} -p_{n}g_{n}+p_{0} & g_{n}<p_{0}/p_{n}\\ 0 & g_{n}\geq p_{0}/p_{n}\; \end{array}\right..$$

Conceptually, the penalty function method involves the insertion of a nonlinear spring between contact surfaces to prevent the derivation of unreasonable results. The penalty factor $p_{n}$ can be interpreted as the stiffness of the spring. In the context of the current computation, it is noteworthy that the variables $g_{n}$ and $T_{n}$ in the equation are restricted by the Kuhn–Tucker conditions to ensure non-negativity. The penalty factor $p_{n}$ and the zero-gap pressure $p_{0}$ are introduced to prevent the occurrence of overclosure at the interface and to correctly calculate the contact pressure $T_{n}$.

#### 2.1.2. Bulk Viscoelastic Damage Model

The small deformation behavior of asphalt concrete facings under static water pressure and gravity follows the linear viscoelastic (LVE) laws. The LVE behavior of the mortars in the closed layer, impermeable layer, and leveling bond layer of asphalt concrete facings can be well described by the generalized Maxwell model. This model can linearly combine the elastic and viscous responses of the material, represented by the elastic modulus and viscosity, respectively, to accurately describe the LVE behavior of the material. The time-dependent modulus of asphalt concrete can be represented by the Prony series in Equation (8b), and the formulations of these equations are given by [37,38]:

(8a) $$\left\{\begin{array}{l} \sigma_{ij}=\sigma_{ij}^{\infty }+\sum_{m=1}^{M}\sigma_{ij}^{m}\\ \varepsilon_{ij}=\varepsilon_{ij}^{\infty }=\varepsilon_{ij}^{m}=\varepsilon_{ij}^{m\cdot el}+\varepsilon_{ij}^{m\cdot vi} \end{array}\right.,$$

(8b) $$E(t)=E_{\infty }+\sum_{m=1}^{M}E_{m}\exp(-\frac{t}{\rho_{m}}),$$

where $\sigma_{ij}^{m}$ and $\varepsilon_{ij}^{\infty }$ are the stress and strain of the single spring element, $\sigma_{ij}^{m}$ and $\varepsilon_{ij}^{m}$ are the stress and strain of the m-th Maxwell branch, and $\varepsilon_{ij}^{m\cdot el}$ and $\varepsilon_{ij}^{m\cdot vi}$ are the elastic strain component and viscous strain component of the m-th Maxwell branch, respectively. $E_{\infty }$ is the long-term equilibrium modulus, and note that it is given in the parent Linear Elastic Materials menu in COMSOL Multiphysics 6.2^®^. *M* is the total number of Maxwell branches, $E_{m}$ is the bulk modulus of the m-th Maxwell branch, and $\rho_{m}$ is the relaxation time of the m-th Maxwell branch. The Prony parameters series are shown in Table 1.

The asphalt concrete impervious facing is a composite material that consists of three layers and exhibits properties similar to concrete. To characterize the evolution of bulk damage during the LVE phase, the well-known Mazars damage model can be employed. This model has been widely applied to materials with concrete-like behavior. It introduces a damage scalar and defines the effective stress as a function of the damage variable, thereby describing the progression of bulk damage within materials. The viscoelastic constitutive relationship of the asphalt concrete impervious facings can be expressed as follows [39]:

(9a) $$\sigma_{ij}^{ve}=(1-D_{M}){\tilde{\sigma }}_{ij}^{ve},$$

(9b) $$D_{M}=\left\{\begin{array}{l} D_{t}(\tilde{\varepsilon })=1-\frac{\kappa_{0}(1-A_{t})}{\tilde{\varepsilon }}-\frac{A_{t}}{\exp[B_{t}(\tilde{\varepsilon }-\kappa_{0})]}\\ D_{c}(\tilde{\varepsilon })=1-\frac{\kappa_{0}(1-A_{c})}{\tilde{\varepsilon }}-\frac{A_{c}}{\exp[B_{c}(\tilde{\varepsilon }-\kappa_{0})]} \end{array}\right..$$

where $\sigma_{ij}^{ve}$ is the nominal viscoelastic stress tensor, ${\tilde{\sigma }}_{ij}^{ve}$ is the effective stress tensor, and $D_{M}\in [0,1]$ is the Mazars damage variable. Moreover, $\kappa_{0}$ represents the initial damage threshold, and $\tilde{\varepsilon }$ is the Mazars equivalent strain; and $A_{t}$, $A_{c}$, $B_{t}$, and $B_{c}$ are parameters measured experimentally.

**Table 1:** Viscoelastic Prony series for the modified asphalt mortar.

Sealing layer ^a^	i	1	2	3	4	5	6
	$\tau_{i}(\;s)$	1 × 10^−13^	2 × 10^−12^	3 × 10^−11^	2.5 × 10^−10^	1.8 × 10^−9^	1.2 × 10^−8^
	$E_{i}(\mathrm{MPa})$	4.44	7.16	10.9	15.7	21.7	28.4
Impervious layer ^b^	i	1	2	3	4	5	6
	$\tau_{i}(\;s)$	1429	148.5	17.57	2.080	0.2015	0.02615
	$E_{i}(\mathrm{MPa})$	2131	1491	1305	826.1	384.3	199.7
Leveling and bonding ^c^	i	1	2	3	4	5	6
	$\tau_{i}(\;s)$	2 × 10^3^	2 × 10^2^	2 × 10^1^	2	2 × 10^−1^	2 × 10^−2^
	$E_{i}(\mathrm{MPa})$	116.6	273.9	597.7	1142.0	1844.0	2499.8

^a^ The data for leveling the cemented layer are from Ref. [40]. ^b^ The data for leveling the cemented layer are from Ref. [41]. ^c^ The data for leveling the cemented layer are from Ref. [42].

### 2.2. Geometric Model, FE Mesh and Constitution Parameters

**Figure 1:**
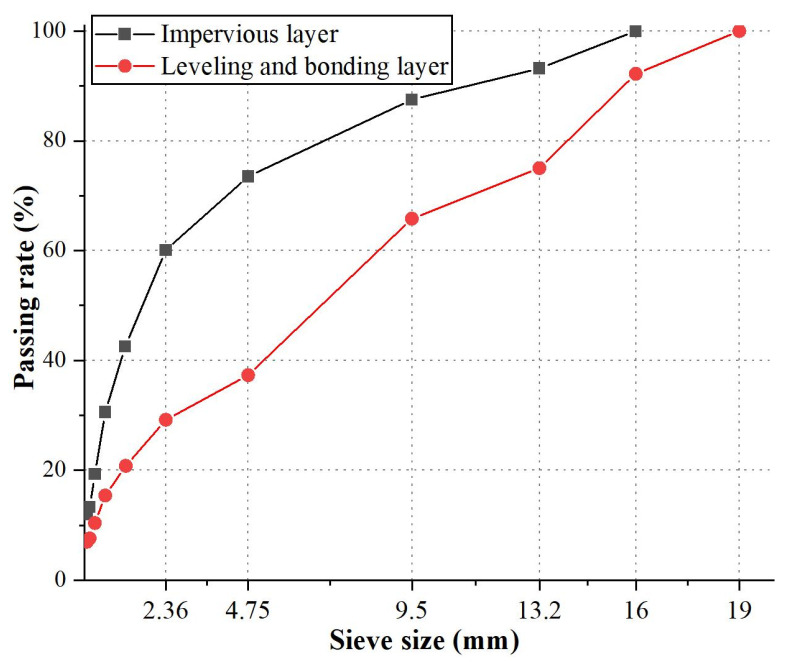
Grading curves.

From top to bottom, the facing comprises sealing, impervious, leveling, and bonding layers. The sealing layer includes modified asphalt mastics, an impenetrable layer of modified asphalt concrete, and the leveling and bonding layer consists of ordinary asphalt concrete. The design mix ratio parameters of each layer can be found in Table 2. And the grading curve is shown in Figure 1.

**Figure 2:**
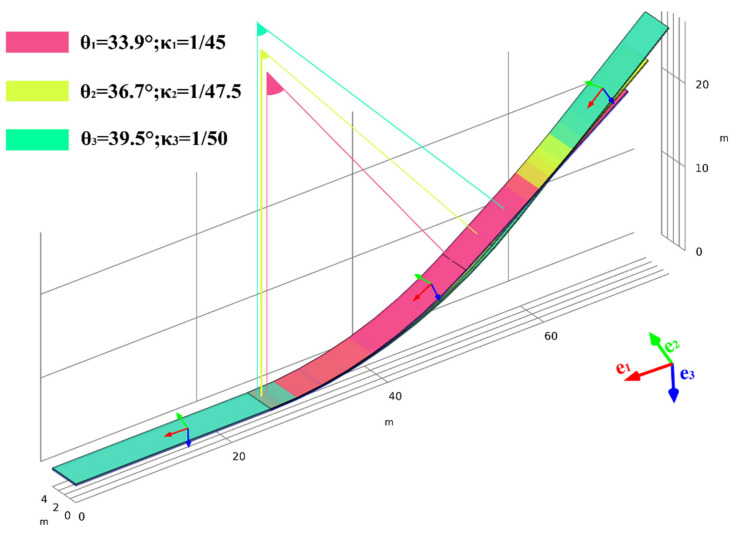
Geometric model of impervious facings.

According to Ref. [43], it is known that the slope ratio of asphalt concrete facings in pumped storage power plants typically ranges from 1:1.4 (33.9°) to 1:1.7 (39.5°). The ogee section corresponds to angles of $\theta_{1}$, $\theta_{2}$, and $\theta_{3}$. Moreover, $\theta_{1}$, $\theta_{2}$, and $\theta_{3}$ represent the curvature $\kappa_{1}=1/45$ m^−1^, $\kappa_{2}=1/47.5$ m^−1^, and $\kappa_{3}=1/50$ m^−1^, as shown in Figure 2. Note that the **e_1_** vector is a consequence slope.

To ensure geometric integrity, cylindrical shells are employed to create three distinct layers, followed by a process of geometric assembly that combines the sealing layer, the impervious layer, the leveling, and the bonding layer interfaces, forming a pair of interfacing layers. It is important to note that the straight plate section of the longitudinal slope is formed by extending the column shell outward from the plane along the slope rather than by splicing. This technique is utilized to prevent any geometric and calculation errors.

**Figure 3:**
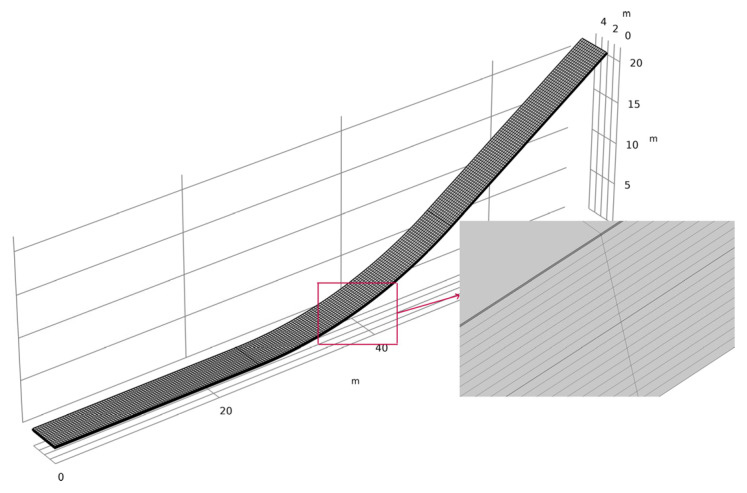
Finite element mesh discretization of the impervious facing.

The mesh is generated through boundary mapping and geometric sweeping. The mesh is refined at the contact interface. Four layers thicken the sealing layer, while the impervious layer and the leveling and bonding layer are each thickened by ten layers. The hexahedral mesh count is 95,088, as shown in the local magnification in Figure 3. During computational simulations, a second-order polynomial function is utilized to define the shape functions of the elements. While this approach may entail increased computational expense, it confers the advantage of enhanced simulation accuracy. In practice, within the COMSOL Multiphysics 6.2^®^ platform, second-order discretization is often employed by default for numerous scenarios. This preference is partly attributed to the fact that many partial differential equations incorporate a predominant second-order derivative term, and the utilization of second-order polynomials facilitates the more precise capture of the characteristics inherent to these derivatives.

Notice that the strength of the two adhesion–decohesion interface models and the strength of the tensile and tensile parameters are considered in four groups: $W_{1}$, $W_{2}$, $W_{3}$, and $W_{4}$ in the following Section 2.3.

For the convenience of calculation, we consider each layer of the asphalt mixture as an isotropic and homogeneous viscoelastic material, taking into account the mechanical properties and design factors and the interface parameters between each layer. According to the literature, the ideal interfacial shear and tensile strength can be achieved when the interlayer spraying amount is 1.8 kg/m^2^. The relevant calculation parameters are listed in the following Table 3.

### 2.3. Random Strength with Weibull PDF

**Figure 4:**
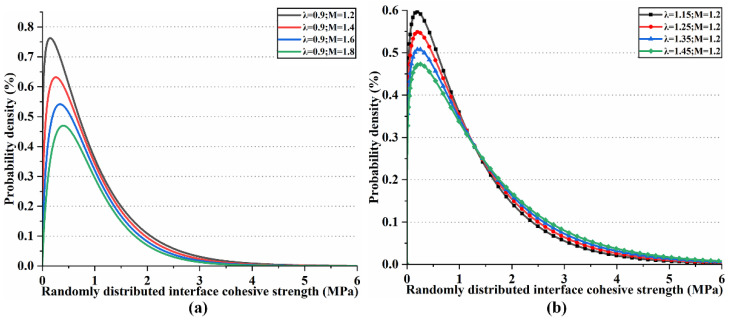
Interface random strength for the (**a**) shear strength and (**b**) tensile strength.

It is worth noting that the spatial random distribution function is used as the shear/tensile strength of the bond layer in accordance with the actual construction conditions. The three-dimensional (3D) Weibull probability density function (PDF) is widely used in engineering and reliability engineering. It usually describes the fatigue life or failure probability of materials or structures in the three-dimensional space. A two-parameter Weibull probability density function distribution, as shown in Figure 4, is implemented in COMSOL Multiphysics 6.2^®^ by using the function definition in Equation (10). The solid line and point plots represent the distribution of the interfacial shear strength and the distribution of the interfacial tensile/compressive strength, respectively. Here, λ=0.9,M=1.2, λ=1.15,M=1.2; λ=0.9,M=1.4, λ=1.25,M=1.2; λ=0.9,M=1.6, λ=1.35,M=1.2; λ=0.9,M=1.8, λ=1.45,M=1.2 are divided into four groups of interfacial tensile/compressive strength distributions, denoted as $W_{1}$; $W_{2}$; $W_{3}$; and $W_{4}$, respectively. Additionally, $W_{1}$ represents λ=0.9,M=1.2 (Weibull parameters for shear strength) and λ=1.15,M=1.2 (Weibull parameters for tensile strength).

**Figure 5:**
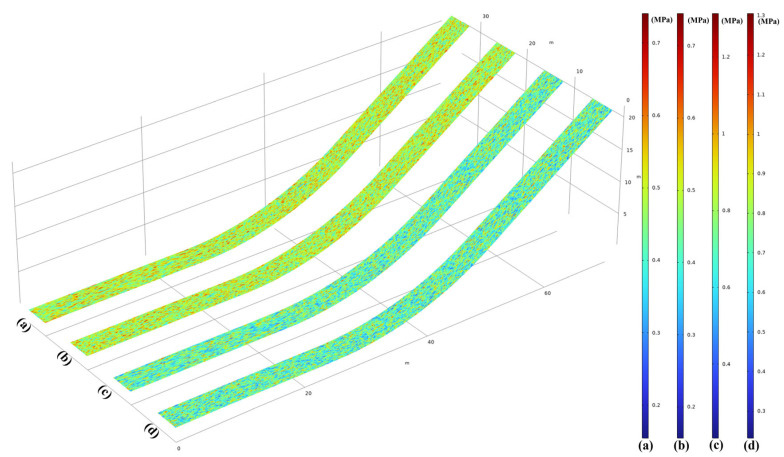
Random tensile/shear strength distribution for sealing layer–impervious layer (**a**,**b**) and impervious layer–leveling and bonding layer (**c**,**d**).

To generate the strength values following the Weibull distribution in space, the boundaries of a geometric figure are defined as the upper and lower limits of a random coordinate interval. Within this coordinate interval, the 1.2 × 10^7^ spatial points (x,y,z) and random intensity values obeying the Weibull distribution are generated by MATLAB, ensuring no missing values on the grid calculation nodes. Subsequently, each random strength in the spatial domain is combined with spatial coordinates to form a dataset $S_{n}=(x,y,z,S_{i})$. Next, interpolation functions $Shear(x,y,z,S_{i})$ and $Tensile(x,y,z,S_{i})$ are created to call upon the data files generated by MATLAB. These functions are invoked when assigning contact interface strength values. A set of interface random strength cloud maps, represented in Figure 5, is plotted. Please note that the geometry used in Figure 2 corresponds to the case of the random strength distribution at curvature $\kappa_{1}=1/45$ m^−1^ in Section 2.2. For other interfaces with different radii/slopes, the generation of random strengths follows the same method, as shown in Figure 5. According to Figure 5a,b, they depict the random distribution of shear strength and tensile strength at the interface of the seal layer and impermeable layer for combination $W_{1}$, respectively, while Figure 5c,d represents the random shear/tensile strength distribution of the impervious layer–leveling and bonding layer interface for combination.

(10) $$f(S_{n},\lambda ,M)=\left\{\begin{array}{ll} \frac{M}{\lambda }{\left(\frac{S_{n}}{\lambda }\right)}^{M-1}\exp\left(-{\left(\frac{S_{n}}{\lambda }\right)}^{M}\right), & S_{n}\geq 0\\ 0, & S_{n}<0 \end{array}\right..$$

where λ and M represent the scale parameter and the Weibull modulus. $S_{n}$ is the random bonding strength of the interface.

### 2.4. Governing Equations and Boundary Conditions

#### 2.4.1. Stress–Strain Field

The inertia effect is neglected in the stress calculation of asphalt concrete facings in this paper. The balance equation can be written as:

(11) $$\sigma_{ij,j}+f_{i}=0,$$

where $f_{i}$ represents the body force, which is the unit weight of the facings in this paper.

For small strain problems, the strain-displacement relationship is:

(12) $$\varepsilon_{ij}=\frac{1}{2}(u_{i,j}+u_{j,i}),$$

where $u_{i}$ is the component of displacement vector u.

#### 2.4.2. Boundary Conditions

To be more in line with the actual working conditions, roller-supported boundary conditions are adopted at the lateral sides of the straight slab and the ogee section.

(13) u⋅n=0.

Due to the complexity of the bedding course materials, it is difficult to find a proper constitutive relation to describe the mechanical behavior. The elastic foundation model adopts the appropriate simplification idea, and its rationality lies in the fact that it can describe the deformation of the foundation better [47]. The foundation is regarded as a set of elastic supports, and the deformation of the foundation is simulated by calculating the foundation reaction force. Therefore, in order to consider engineering practice, it is common to assume that the support of the lower part of the dam and reservoir basin is on an elastic basis, that is, there are uniformly distributed springs at the bottom of the impervious facings. When an external force is applied to the spring, it undergoes small deformation and stores elastic potential energy. According to the principle of virtual work, the virtual work produced by these small displacements must be zero because the system is still in equilibrium during these small displacements. The equations are as follows:

(14a) $$\delta W=\int_{A}\left(f_{s}+f_{1}+f_{v}\right)\cdot \delta udA,$$

(14b) $$\left\{\begin{array}{l} f_{s}=-K\cdot (u-u_{0})\\ f_{1}=-i\eta K\cdot (u-u_{0})\\ f_{v}=-\mu \cdot (\dot{u}-{\dot{u}}_{0}) \end{array}\right..$$

where $f_{s}$ is the force per unit, $f_{1}$ is the loss factor, and $f_{v}$ is the viscous damping. K, i, η, and μ represent the stiffness matrix, imaginary unit, spring loss coefficient, and matrix representing the viscosity, respectively.

#### 2.4.3. Boundary Load

**Figure 6:**
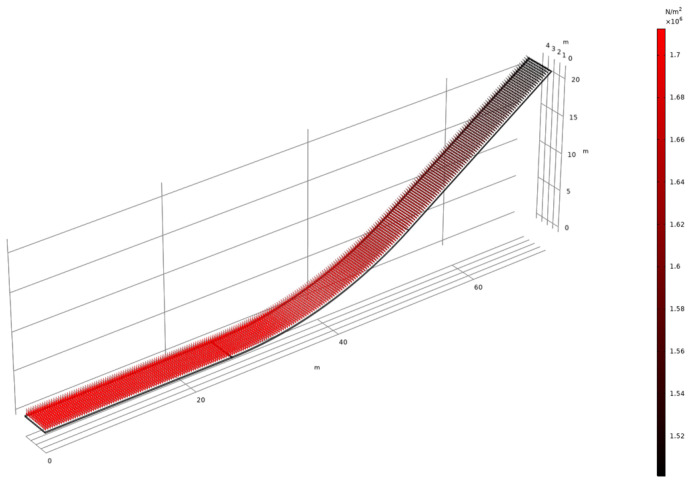
The static water pressure of impervious facings.

The loading mode of the facings is selected to use an exponential function to avoid sudden stress or strain changes for improving the convergence of the calculations. The equation is as follows:

(15) $$P=P_{0}\left(1-\exp\left(-\frac{t}{t_{0}}\right)\right)$$

where $P_{0}$ is selected as 1.5 MPa [43] and $t_{0}$ is set as 3 s. The loading mode and load size are related to the height and time of the facing. The load diagram as shown in Figure 6. Please note that we have taken into account the effect of gravity and it is not represented in the figure.

## 3. Model Verification by Direct Shear Tests

**Figure 7:**
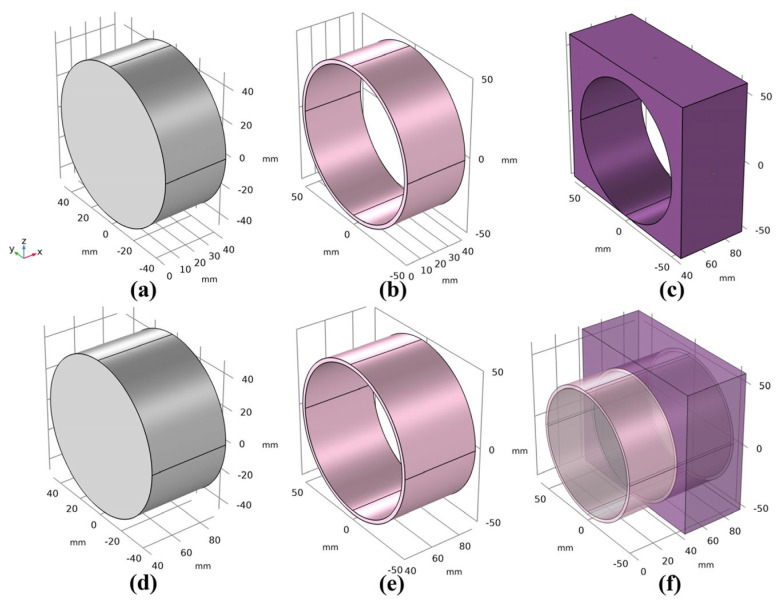
Simplified principle diagrams of the direct shear test. (**a**) is the wearing layer with a diameter of 97 mm and a thickness of 40 mm, (**b**) is the steel split ring, (**c**) is the vertical supporting plate, (**d**) is the binder layer with a diameter of 97 mm and a thickness of 50 mm, (**e**) is the steel cylinder welded on the vertical supporting plate, and (**f**) is the simplified principle diagram of the direct shear test.

In this section, starting from the interlayer direct shear tests with the normal load of asphalt concrete roads [11], the reliability of the CZM model proposed in this paper is verified and then it is extended to asphalt concrete impervious facings and their ogee sections. The specimens and equipment for the direct shear tests between asphalt concrete layers with q normal load are shown in Figure 7a–f, where (a) is the wearing layer with a diameter of 97 mm and a thickness of 40 mm, (b) is the steel split ring, (c) is the vertical supporting plate, (d) is the binder layer with a diameter of 97 mm and a thickness of 50 mm, (e) is the steel cylinder welded on the vertical supporting plate, and (f) is the simplified principle diagram of the direct shear test.

To ensure consistency between the numerical simulation and the direct shear test under a normal load, it is necessary to constrain the boundary conditions of the specimen. In the specimen in Figure 7d, the upper and lower boundaries are considered to be fixed, resulting in a fixed-constrained boundary condition:

(16) u=0.

During the loading process of Figure 7b, there is a uniform downward movement in the vertical direction. Therefore, the upper and lower surfaces of Figure 7a should be treated as limited to *z*-axis displacement, with each node’s velocity specified as (0,0,−v). In addition, the rate should be strictly set to 0.2 mm/s, according to the given experimental conditions.

**Figure 8:**
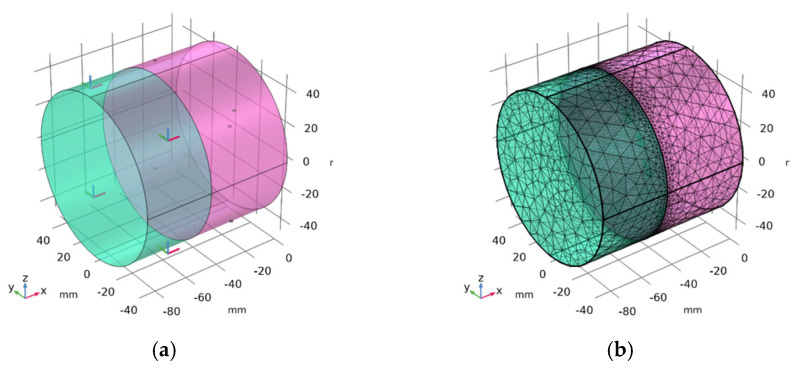
(**a**) Pull-off model diagram. (**b**) Mesh of the model diagram.

It is necessary to control the loading and boundary conditions used in calculations consistent with those used in experiments [11]. Based on laboratory conditions, a viscous coating is sprayed on Figure 7a,d, and adhesion is achieved through the contact interface. The loading process in the indoor experiment involved the vertical actuator pushing down on the steel cup (Figure 7b) at 0.2 mm/s until the displacement recorded by the sensor reached 12 mm. The simplified calculation model is shown in Figure 7f. In addition, to ensure the precision of the calculation, the sample mesh is set to be finer, with a total of 71,741 free tetrahedral elements (see Figure 8b), and the average mesh quality is 0.65.

**Figure 9:**
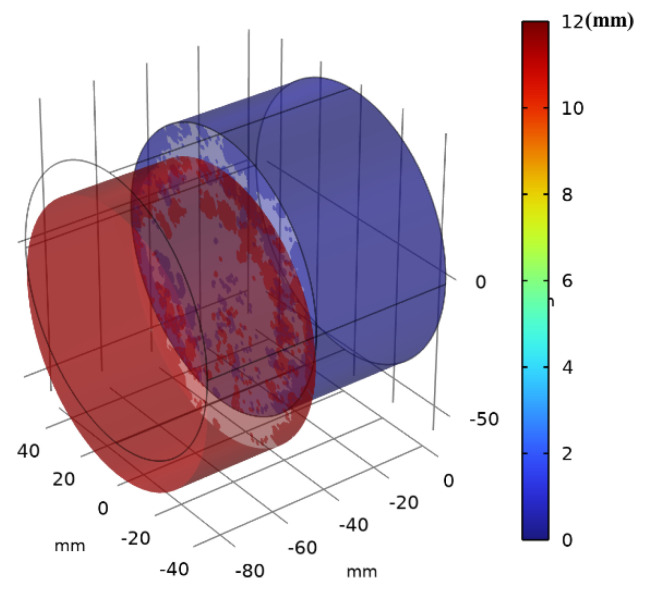
The simulated displacement after 12 s under vertical loading of the interface direct shear test.

**Figure 10:**
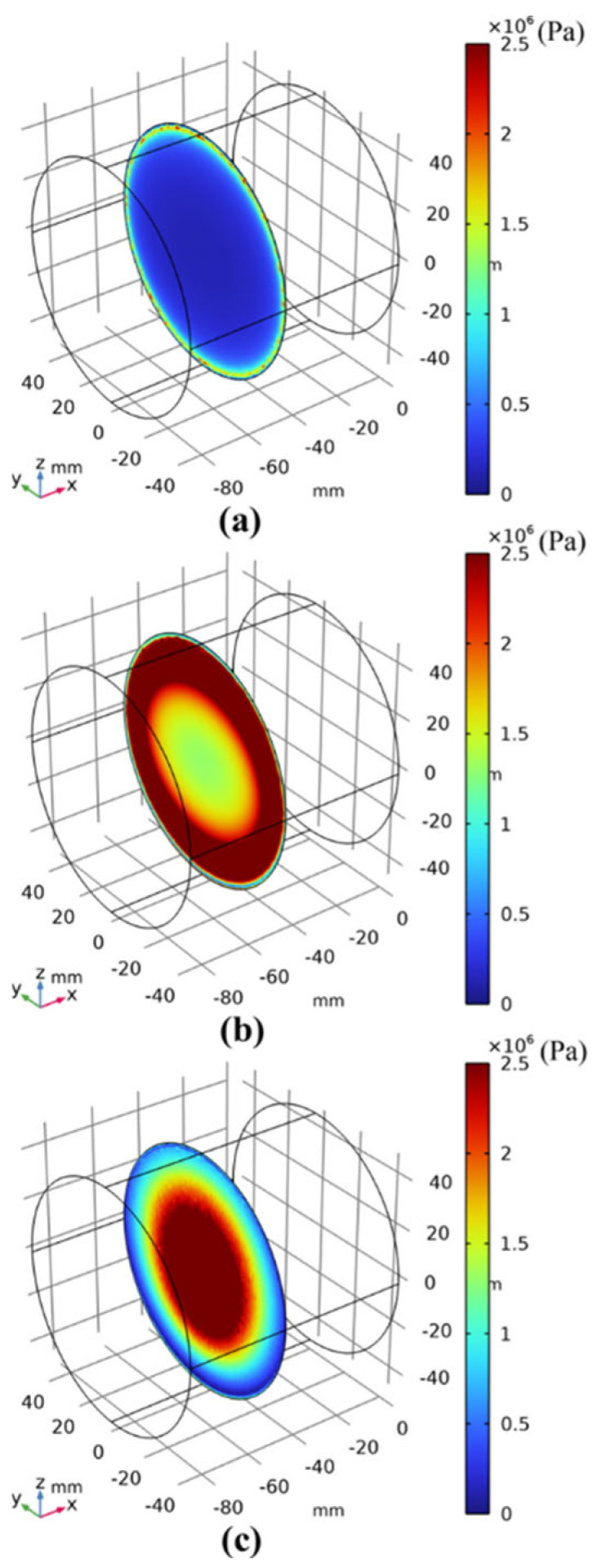
Interfacial shear stress distribution after 12 s under vertical loading of the interface direct shear test. (**a**–**c**) is the result when the vertical drive is depressed for 1 s, 10 s, and 20 s, respectively.

**Figure 11:**
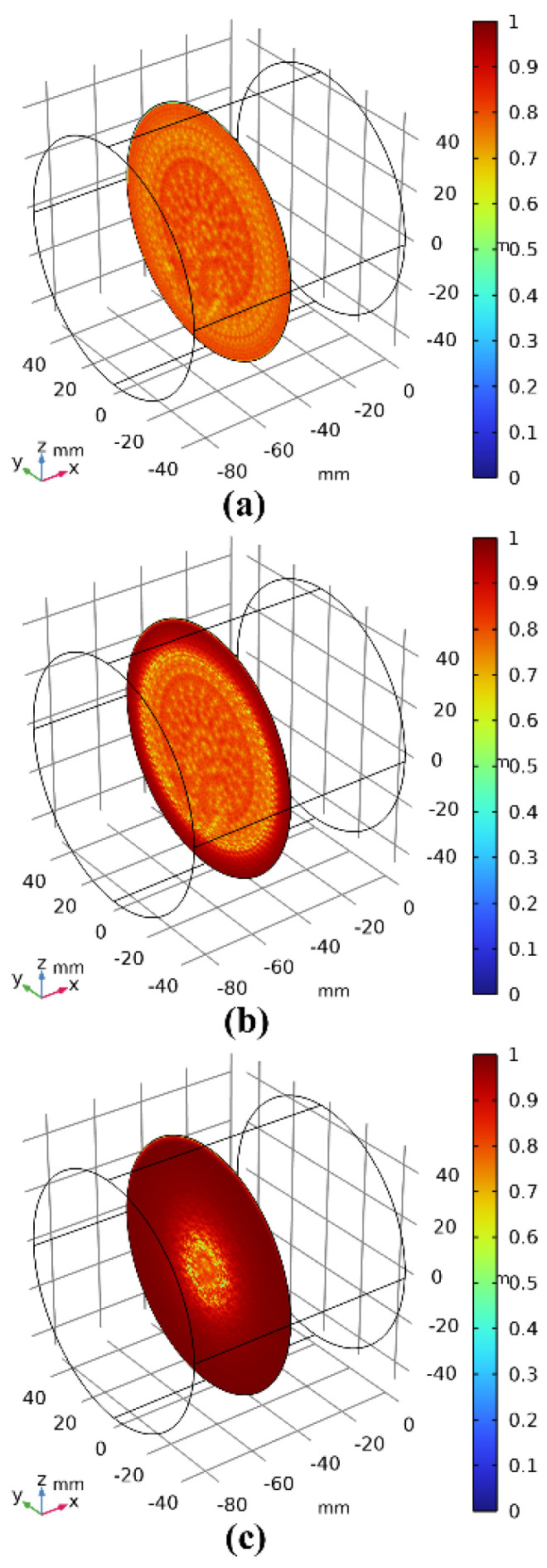
Interfacial damage distribution after 12 s under vertical loading of the interface direct shear test. (**a**–**c**) is the result when the vertical drive is depressed for 1 s, 10 s, and 20 s, respectively.

**Figure 12:**
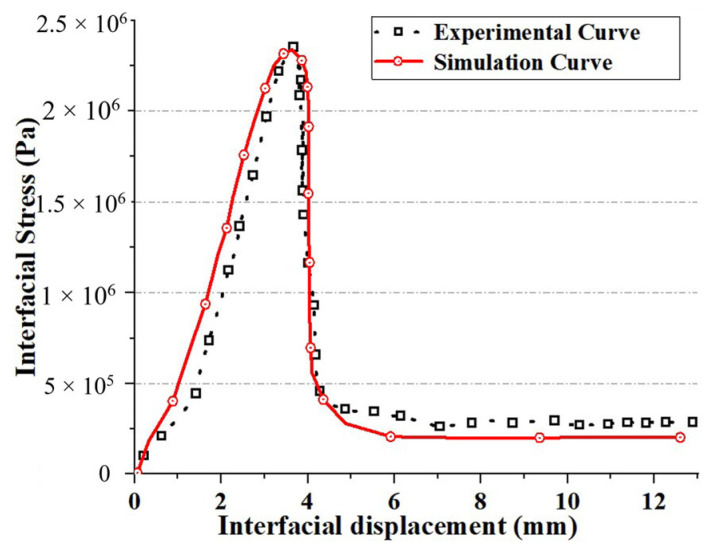
Comparing the verification curve of interfacial shear stress with the experimental curve [11].

Figure 9 shows the image obtained after pushing the vertical driver down by 12 mm (t = 60 s). Since the decohesion damage in this calculation model is determined by displacement, the displacement mismatch of the interface layer during the process of pushing the vertical driver down can cause interface damage. When the interface damage value reaches 1, according to the literature [48], it can be determined that the interface has failed. Figure 10a–c show the results when the vertical driver is pushed down for 1 s, 10 s, and 20 s, respectively (hereafter, the same applies to the figures mentioned later). Figure 11a–c represent the interface failure conditions at different times. When the damage distribution around the interface expands inward, it eventually leads to interface decohesion, as can be seen in Figure 11a–c. The shear stress distribution can be seen in Figure 12, which shows a significant drop in shear stress when the interface fails completely after 20 s. This conclusion is completely consistent with the literature [11], indicating that the cohesive zone damage model based on displacement can correctly simulate the interface shear test of asphalt concrete layers. Although there are still slight differences in the results, this may be due to the effects of voids and defects in the experimental samples not being considered.

## 4. Results and Discussion

Based on the interface model we proposed above, we extend its application to asphalt concrete impervious facings. The geometric model of the impervious facing is categorized into three cases: $\theta_{1}$ and $\kappa_{1}$; $\theta_{2}$ and $\kappa_{2}$; $\theta_{3}$ and $\kappa_{3}$. Simultaneously, we analyze the distribution of the interface stress and strain, and we explore the influence of the ogee section curvature on the interface stability. It is important to note that the coordinate system used in this section is a local curvilinear coordinate system, as shown in Figure 2.

### 4.1. Shear Stress and Strain Analysis of Interfaces

The impervious facing can be simplified to a three-layer composite structure on an elastic foundation, as depicted in Figure 2. It experiences the main loads due to static water pressure and the counteracting force from the elastic foundation, which approximates the supporting action from the cushion layer and the remaining parts of the reservoir basin. This approximation represents the supporting action.

The pressure load is transmitted through the interfaces: the one between the sealing layer and the impervious layer (the first interface), and the other between the impervious layer and the leveling layer, which is part of the bonding layer interface (the second interface). Damage to these interfaces can lead to a reduction in impervious capability and an increase in operation and maintenance costs.

**Figure 13:**
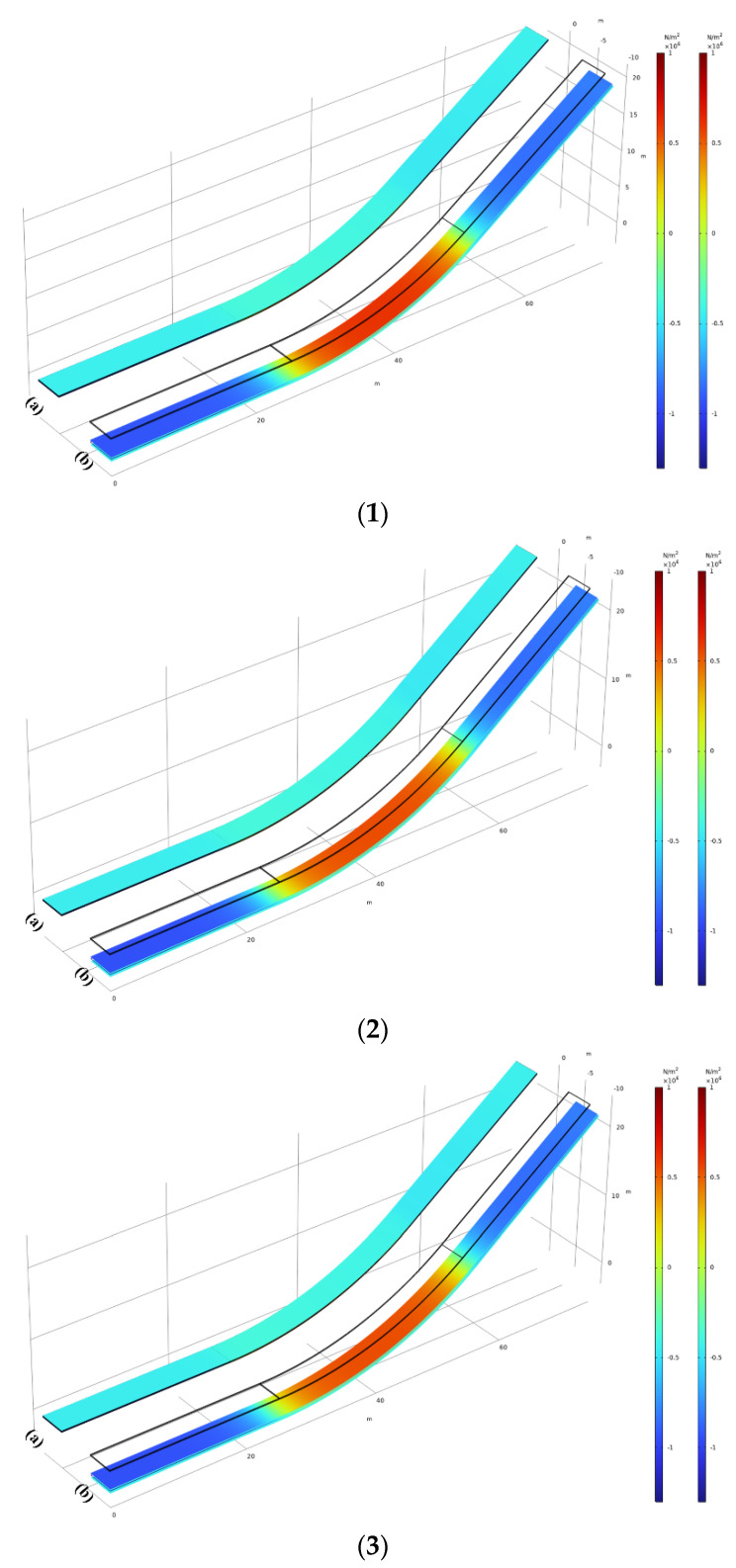
(**1**) Global principal stress 1 of (**a**) front view of the facing and (**b**) back view of the facing for $\kappa_{1}$. (**2**) Global principal stress 1 for (**a**) front view of the facing and (**b**) back view of the facing for $\kappa_{2}$. (**3**) Global principal stress 1 for (**a**) front view of the facing and (**b**) back view of the facing for $\kappa_{3}$.

The relatively small thickness of the three layers in the ogee section of the facing is the main reason why the front of the impervious facings is primarily influenced by compressive stress, while the back is influenced by tensile stress. As shown in Figure 13(1–3), where (1), (2), and (3) represent the conditions for $\kappa_{1}=1/45$ (m^−1^), $\kappa_{2}=1/47.5$ (m^−1^), and $\kappa_{3}=1/50$ (m^−1^), respectively, a larger curvature predominantly causes greater compressive stress on the front and tensile stress on the back of the facings.

**Figure 14:**
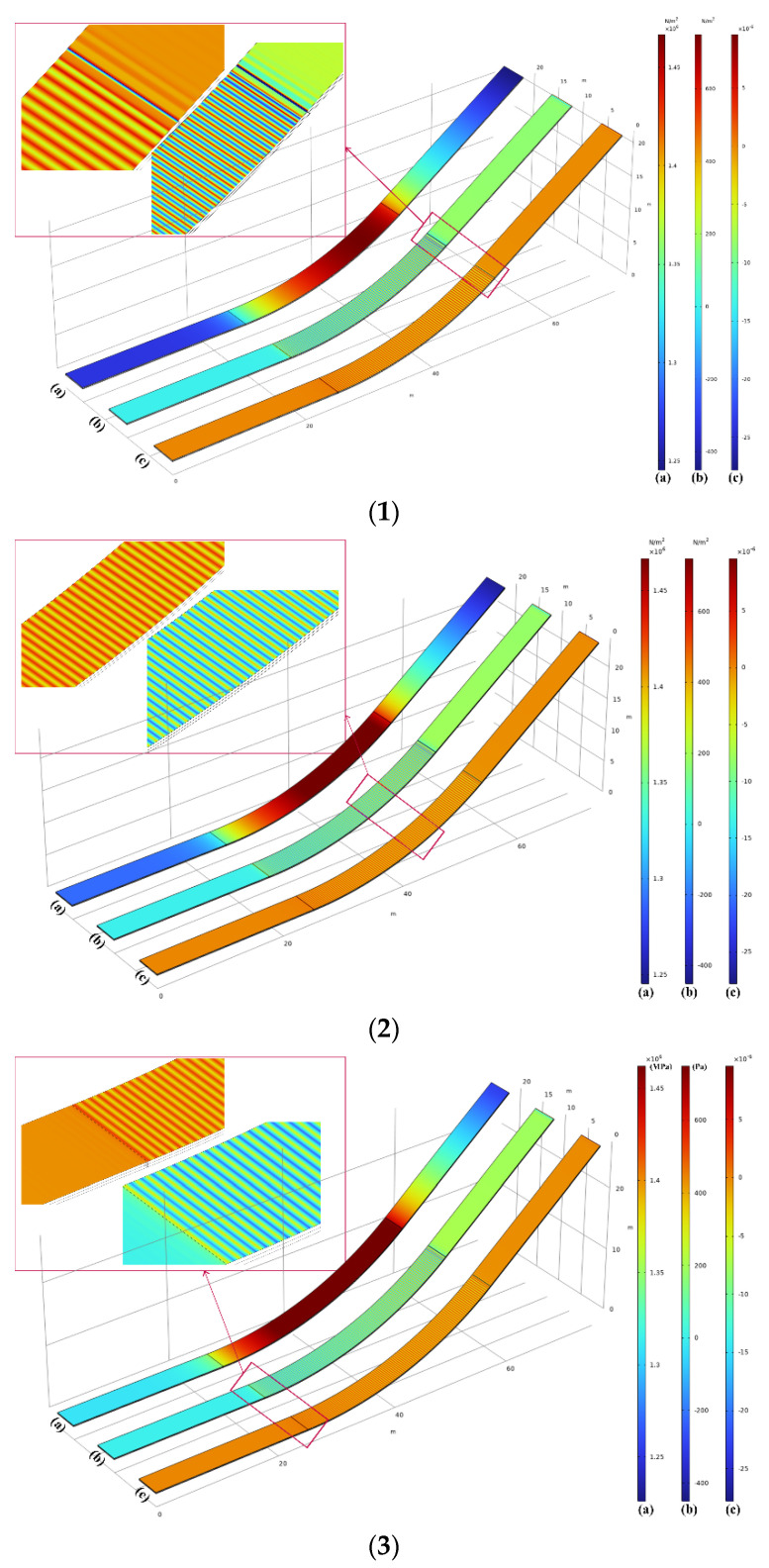

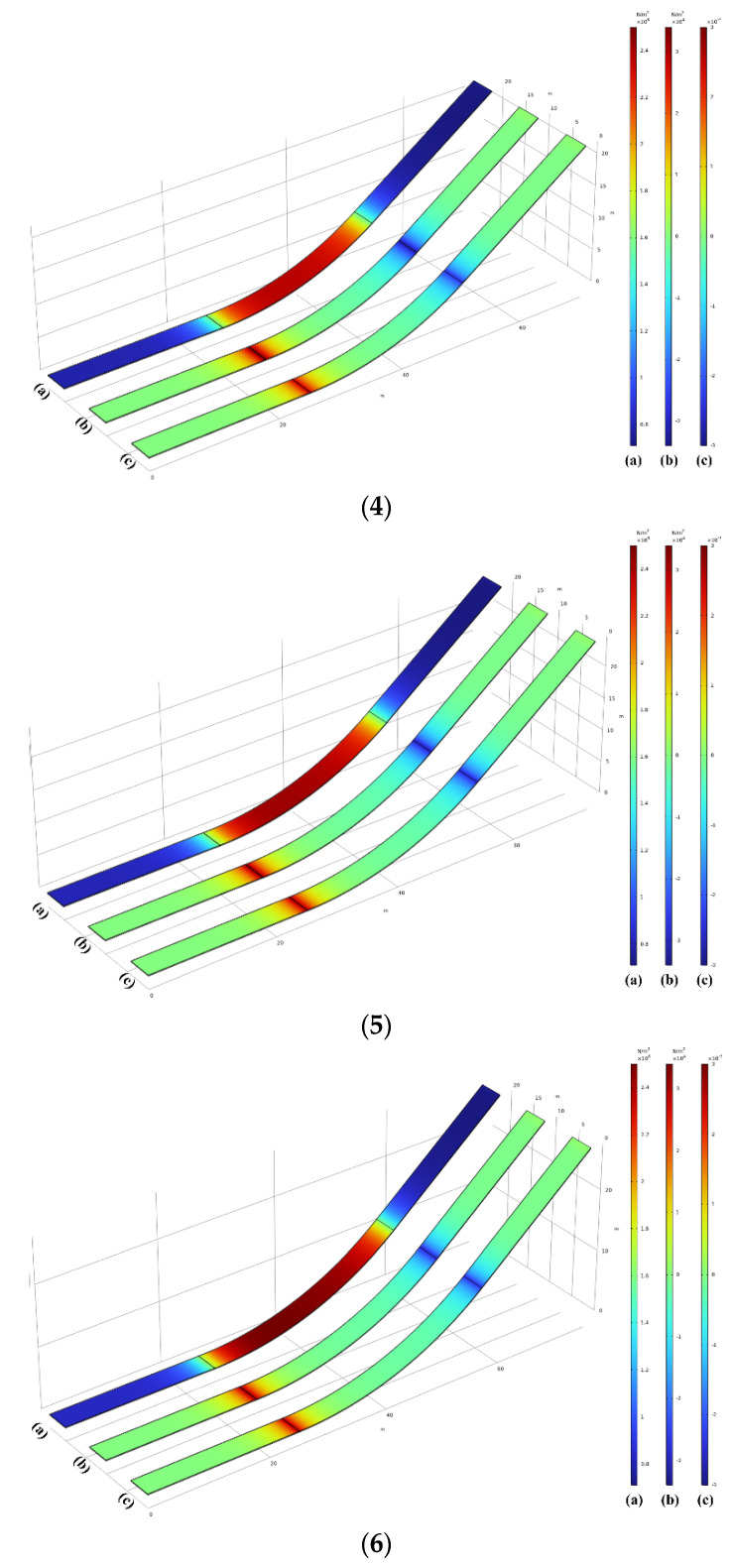
(**1**) Interface 1: (**a**) maximum shear stress, (**b**) local shear stress, and (**c**) local shear strain for $\kappa_{1}$. (**2**) Interface 1: (**a**) maximum shear stress, (**b**) local shear stress, and (**c**) local shear strain for $\kappa_{2}$. (**3**) Interface 1: (**a**) maximum shear stress, (**b**) local shear stress, and (**c**) local shear strain for $\kappa_{3}$. (**4**) Interface 2: (**a**) maximum shear stress, (**b**) local shear stress, and (**c**) local shear strain for $\kappa_{1}$. (**5**) Interface 2: (**a**) maximum shear stress, (**b**) local shear stress, and (**c**) local shear strain for $\kappa_{2}$. (**6**) Interface 2: (**a**) maximum shear stress, (**b**) local shear stress, and (**c**) local shear strain for $\kappa_{3}$.

Subsequently, the shear strain experienced by the facing is mainly oriented in the downslope direction, as shown in Figure 14(1–3). Here, (1), (2), and (3) correspond to the conditions for cases $\kappa_{1}$, $\kappa_{2}$, and $\kappa_{3}$ the same as in Figure 13. Simultaneously, they also illustrate the maximum shear stress, the local shear stress, and the local shear strain along the first-layer interface, respectively. Note that in Figure 14(1–3), the labels (a), (b), and (c) all pertain to outcomes at the first-layer interface, and in Figure 14(4–6), the labels (a), (b), and (c) represent results at the second-layer interface.

The maximum shear stress along the first-layer interface of the ogee section predominantly distributes on the upper side of the ogee curve, as shown in Figure 14(1–3)(b). The maximum shear stress in the downslope direction at the first-layer interface is smaller than that at the second-layer interface. However, localized shear stress folding occurs along the first-layer interface, as indicated in Figure 14(1–3)(b). A rippling deformation phenomenon emerges within the region of the first-layer interface, which is more common in shell structures but less likely in relatively thicker structural layers, such as the second-layer interface. This can be seen in the shear stress and strain distribution at the second-layer interface in Figure 14(4–6). Therefore, the first layer of the interface has an alternating distribution of positive and negative strain/stress values, which appear in the form of stripes, as observed in Figure 14(1–3)(b,c). Regions exhibiting this distribution of stress oscillations are prone to local instability, leading to pronounced wrinkling on thin facings, aligning with the conclusions in Ref. [31]. It can be observed that the interface wrinkles vanish at the junction between the straight section and the ogee section. This phenomenon occurs because, as the curvature approaches infinity, the instability strain causing the wrinkles tends toward zero, as explained in Ref. [32].

### 4.2. Damage Analysis of Interface

When the impervious facing is subjected to positive pressure and gravity, it experiences two main damage modes, the adhesion–decohesion damage and compression–shear damage, at its two-layer interfaces. It should be pointed out that the former mode is described by the CZM and the latter is one branch of the Mazars damage. The stress distribution within the interface bonding layer becomes uneven, with stress in certain regions surpassing the strength limit of the interface elements. This leads to a weakening of the adhesion forces, causing the adhesion failure between the two layers and the occurrence of interface delamination. Interfacial shear damage involves relative sliding within the material layer. The stress concentration occurs at the facings–ogee section interface. This sliding weakens the adhesion stress of the interface and leads to the interface debonding damage. The combined impact of these two types of damage leads to a decrease in the impermeability performance of the impermeable facing and contributes to structural degradation [49,50,51].

**Figure 15:**
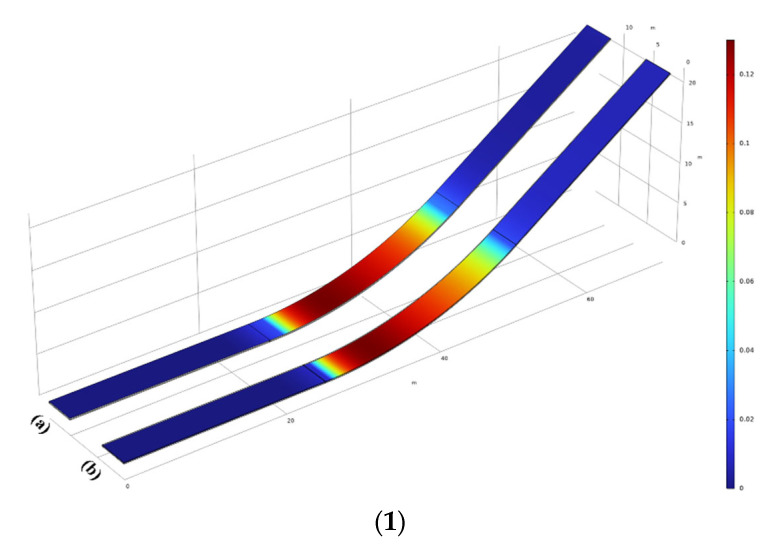

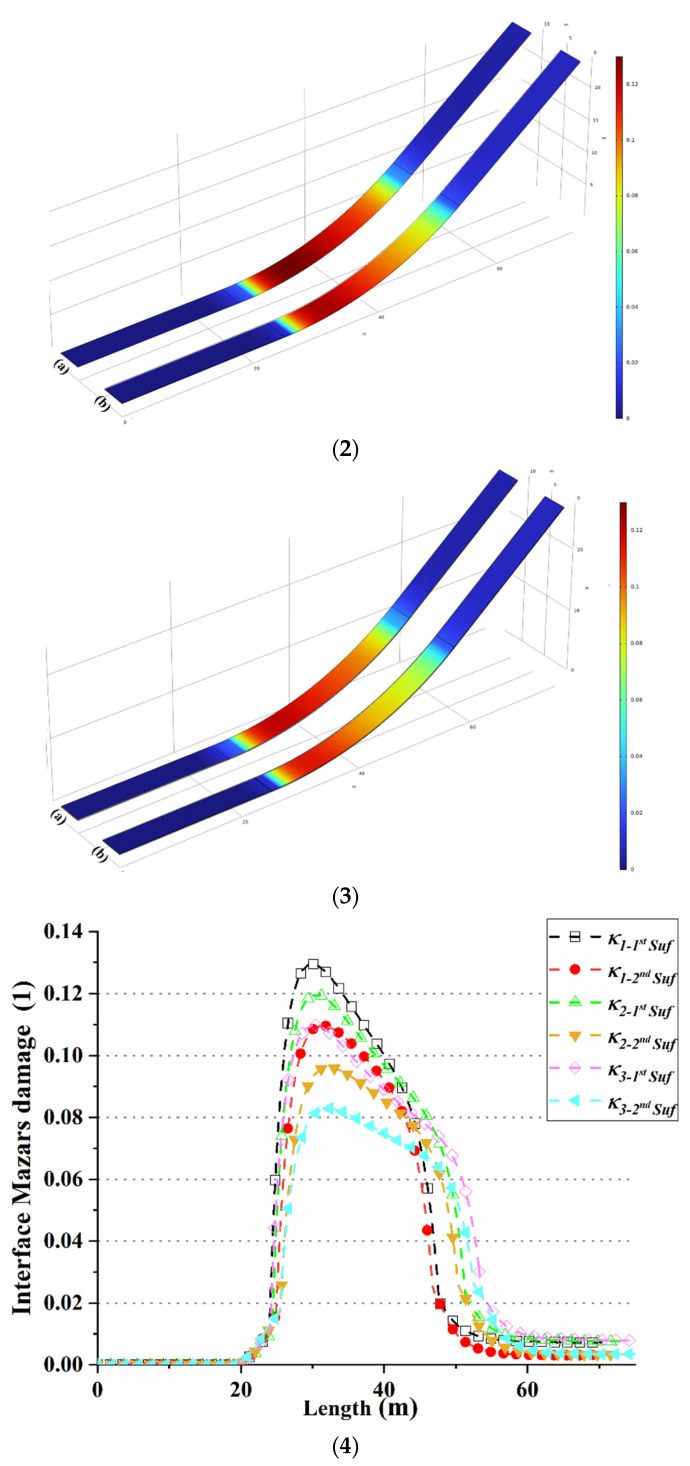
(**1**) The Mazars damage of (**a**) interface 1, and (**b**) interface 2 for $\kappa_{1}$. (**2**) The Mazars damage of (**a**) interface 1, and (**b**) interface 2 for $\kappa_{2}$. (**3**) The Mazars damage for (**a**) interface 1 and (**b**) interface 2 for $\kappa_{3}$. (**4**) The Mazars damage diagram of different curvatures.

The compression–shear damage (i.e., the Mazars damage) zone at the interface primarily exists within the ogee section’s first-layer interface, as shown in Figure 15(1–3)(a), and the second layer of the interface is mainly the tensile damage area, as shown in Figure 15(1–3)(b). In the interface, the closer the area is to the left reverse arc, the larger the compression shear damage area is. The compression–shear damage in the first-layer interface is slightly higher than that in the second-layer interface. By subdividing the abscissa into the smallest grid size (denoted as x), ensuring the accuracy of the extracted data points, and calculating the average damage value within the minimum unit size interval (denoted as y), the double-layer interface damage curve under different curvatures is obtained. The hollow point line graph represents the results of the first-layer interface, while the solid point line graph represents the results of the second-layer interface, as shown in Figure 15(4). The value of the interfacial compression–shear damage increases sharply between the linear section and the ogee section. This is due to the direct conversion of the curvature between the elliptical and straight section 1/∞. The change in the loading direction will lead to a sudden change in the stress state and damage distribution at the interface.

According to Figure 15, it can be observed that compression–shear damage primarily occurs at the interface of the ogee section. Interfaces at the ogee section with larger curvatures exhibit greater damage values compared to those with smaller curvatures. Additionally, the first-layer interface experiences higher levels of damage under the compression–shear stress state compared to the second-layer interface.

**Figure 16:**
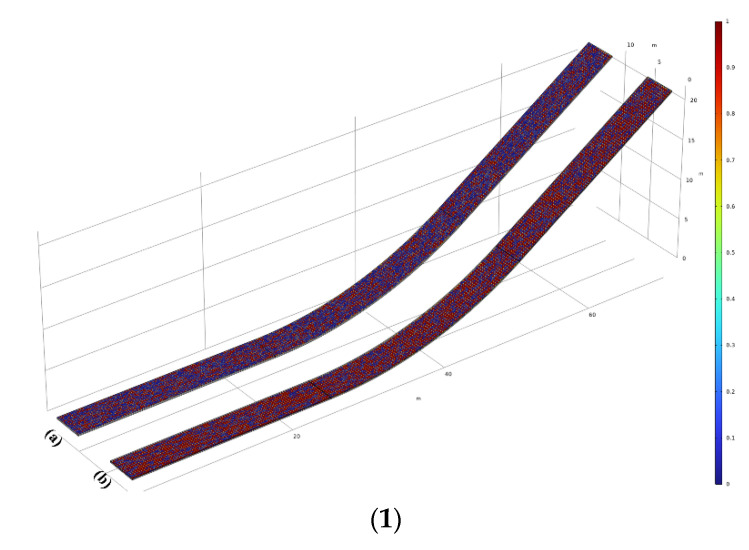

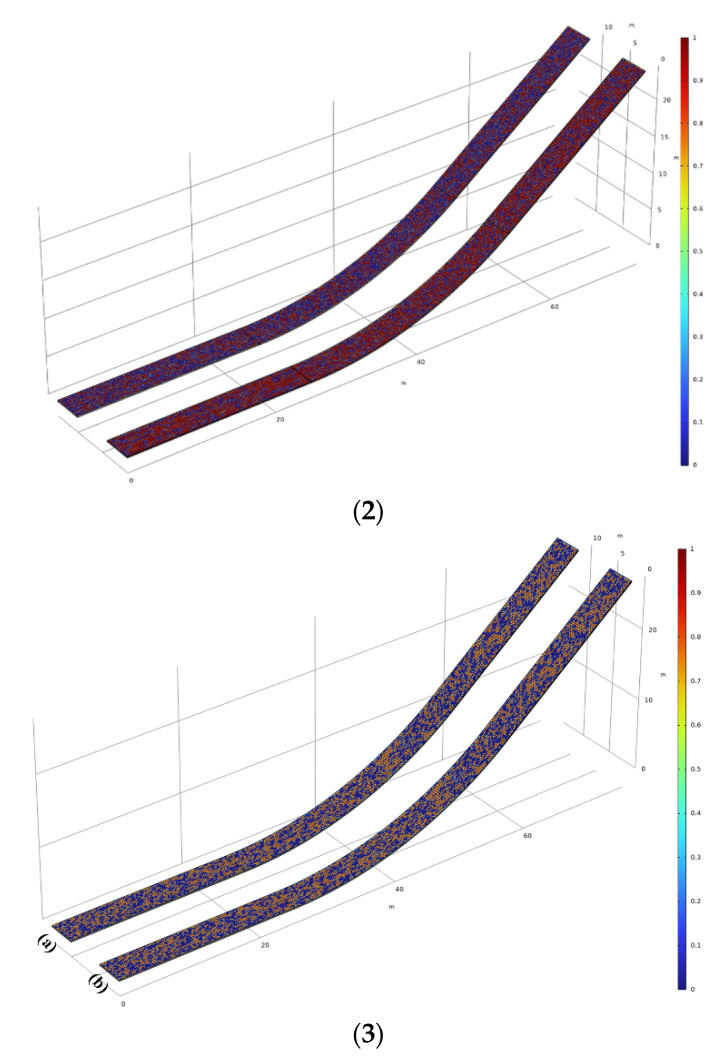
Adhesion and decohesion damage of (**a**) interface 1, and (**b**) interface 2. (**1**) Adhesion and decohesion damage for $\kappa_{1}$. (**2**) Adhesion and decohesion damage for $\kappa_{2}$. (**3**) Adhesion and decohesion damage for $\kappa_{3}$.

**Figure 17:**
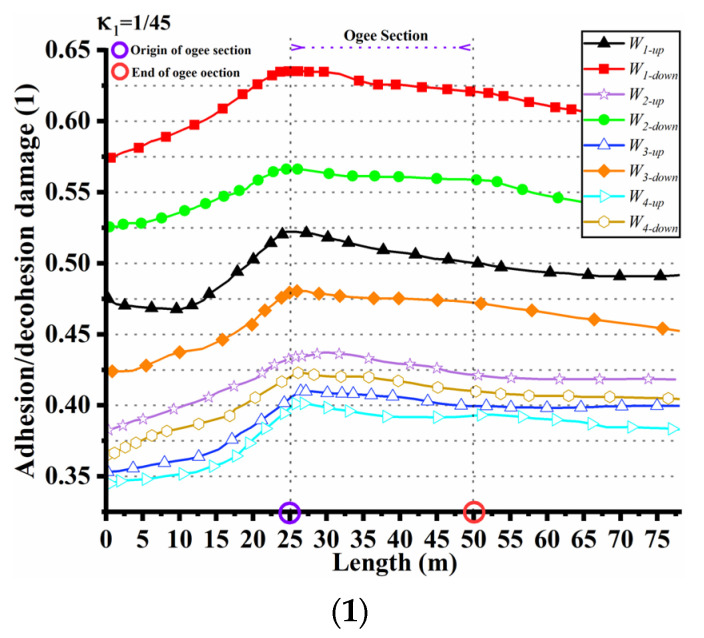

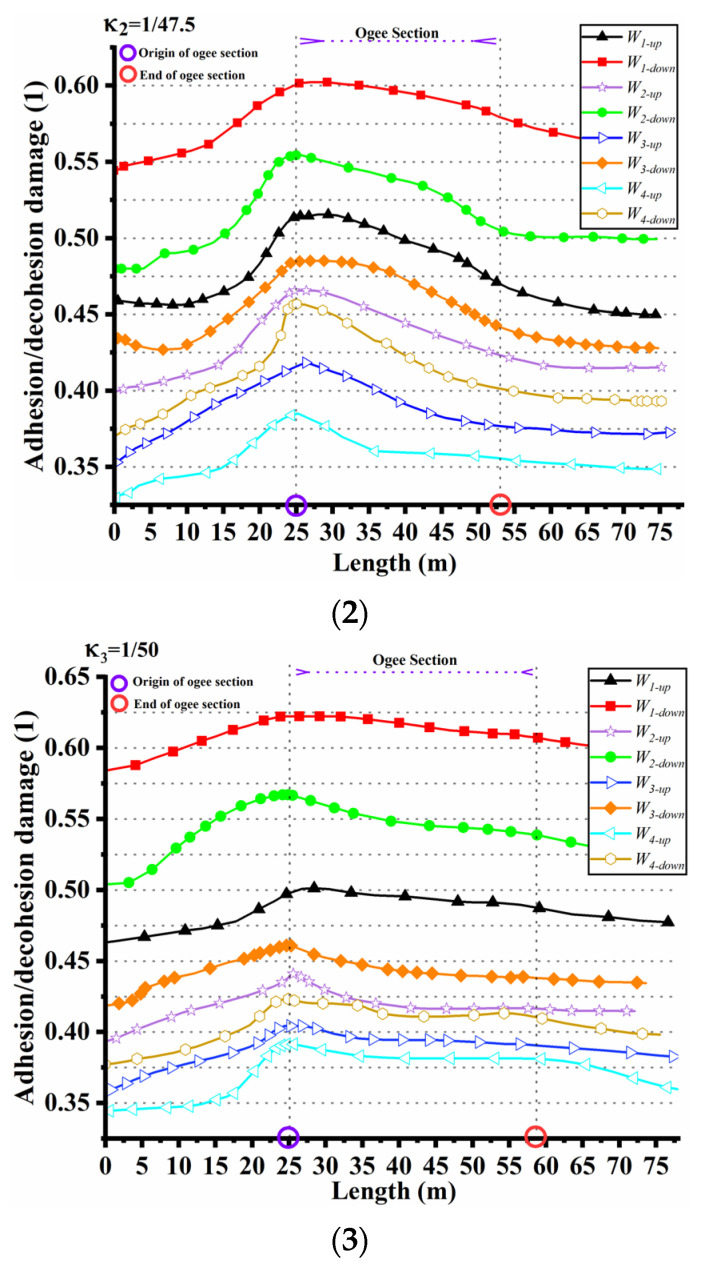
(**1**) Adhesion and decohesion damage for $\kappa_{1}$. (**2**) Adhesion and decohesion damage for $\kappa_{2}$. (**3**) Adhesion and decohesion damage for $\kappa_{3}$.

Analysis of Figure 16(1–3) reveals the presence of adhesion–decohesion damage at the interfaces between the first and second layers. Through the processing of the associated damage data, we have derived the interface damage profiles under a range of curvature conditions, as demonstrated in Figure 17(1–3). These profiles indicate an initial gradual ascent in the adhesion–decohesion damage on the left side of the ogee section, culminating in a peak at the section’s summit, beyond which the damage decrements progressively. This observed pattern of damage progression closely corresponds to the predictive trajectory of the Mazars damage model. It is particularly observed that an augmented curvature within the ogee section is correlated with an escalated magnitude of adhesion–decohesion damage.

Contrary to the Mazars damage pattern, our findings indicate that the adhesion–decohesion damage at the second-layer interface is more pronounced than at the first-layer interface. This reversal can be attributed to the structural differences between the layers: the first layer, serving as a sealing layer, is relatively thinner compared to the second layer, which acts as an impervious layer. The reduced thickness of the first layer predisposes it to buckling instability prior to the second layer. Consequently, within this layer, decohesion damage induced by shear stress is not the predominant factor. In scenarios involving compression–shear damage, the adhesion–decohesion damage, however, assumes higher values, particularly in the context of interlayer sliding failure modes. For impervious facing structures, this dynamic implies that while compression–shear damage is the predominant factor affecting the first layer, adhesion–decohesion damage assumes a secondary role. Conversely, at the second-layer interface, adhesion–decohesion damage becomes the primary concern, overshadowing the effects of compression–shear damage.

Considering the significant impact of the random distribution of the interface strength on analyzing the interface damage, different combinations of Weibull modulus and characteristic strength are better with actual construction conditions. According to Figure 17(1–3), the four sets of random strength values are labeled as $W_{1}$, $W_{2}$, $W_{3}$, and $W_{4}$ specify the adhesion–decohesion damage for the Weibull parameter $W_{1}$ as $D_{W_{1}}$. The adhesion and decohesion damage tendency of the interface is $D_{W_{1}}>D_{W_{2}}>D_{W_{3}}>D_{W_{4}}$. As the Weibull modulus *M* increases, the variation in the random interface strength also increases, leading to improved interface bonding performance and less pronounced damage. Conversely, a smaller Weibull modulus results in smaller variations in the random interface strength, lower interface bonding performance, and more noticeable damage. The Weibull parameter of this model can also be used to evaluate the damage distribution of the cemented surface of the anti-seepage facings under different curvatures of the impervious facings, providing a valuable reference for the design of the impervious facings.

## 5. Conclusions

This study proposes a three-dimensional finite element interface mixed damage model for asphalt concrete impervious facings. The model consists of two sub-models: one is an interface cohesive zone damage model, which simulates adhesion and debonding phenomena, and the other is a bulk Mazars damage model, which characterizes material degradation. Before delving into in-depth research, we first validated the feasibility of the cohesive zone model (CZM) based on indoor direct shear tests in simulating interface adhesion and debonding damage through finite element simulation. Subsequently, we applied this model to three instances of hydropower asphalt concrete impervious facings with different curvatures in pumped hydro storage projects. During the calculation process, the random distribution of the interface strength is considered, and especially in the calculation of the interface damage, the combined effects of shear damage and adhesion-debonding damage are comprehensively considered. Defects that may occur during the construction process, such as uneven mixing, porosity, and cracks, which can affect the bonding performance of the interface. The potential impact of these defects on the material’s fatigue life can be assessed through the distribution of the Weibull random moduli.

Under the mixed damage modes of compression–shear and adhesion–debonding, the instability of the interface is particularly evident, especially in models with larger curvatures. The interface mixed damage model established in this paper can not only be used to evaluate the stability of the two interfaces of the impervious facings but also provides important theoretical support for the design and construction of asphalt concrete impervious facings. To further develop this model, other practical factors such as temperature fluctuations, material aging, and the continuous impact of long-term loading need to be considered. To ensure the practicality and reliability of the model, further research is needed to better consider the inherent randomness of the actual engineering environment.

## Figures and Tables

**Table 2:** Asphalt mixture sieving rate.

Impervious layer	Sieve size (mm)																			
	16		13.2		9.5		4.75		2.36		1.18		0.6		0.3		0.15		0.075	
	Passing rate (%)																			
	100		93.2		87.5		73.5		60.1		42.5		30.6		19.3		13.3		12.0	
Leveling and bonding layer	Sieve size (mm)																			
	19	16		13.2		9.5		4.75		2.36		1.2		0.6		0.13		0.15		0.075
	Passing rate (%)																			
	100	92.2		75.0		65.8		37.3		29.2		20.8		15.4		10.4		7.6		7.0

**Table 3:** Calculation parameters.

	Young’s Modulus (GPa)	Tensile Strength (MPa)	Compressive Strength (MPa)	Density (g/cm^3^)	Poisson’s Ratio (1)
Sealing layer ^a^	0.086	7.6	2.61	1.73	0.2
Impervious layer	1.30	1.37	5.52	2.44	0.25
Leveling and bonding layer ^b^	1.80	1.2	4.82	2.31	0.32
	Tensile strength (MPa)			Shear strength (MPa)	
Interface ^c^	0.56 ^1^			0.936 ^2^	
Interface (Random)	$Tensile(x,y,z,S_{i}^{2})$			$Shear(x,y,z,S_{i}^{1})$	

^a^ The data for sealing layer are from Ref. [44]. ^b^ The data for leveling and bonding layer are from Ref. [45]. ^c1^ The tensile strength for leveling the cemented layer is from Ref. [27]. ^c2^ The shear strength for leveling the cemented layer is from Ref. [46].

## Data Availability

No new data were created or analyzed in this study. Data sharing is not applicable to this article.
